# Antidiabetic Potential of Black Elderberry Cultivars Flower Extracts: Phytochemical Profile and Enzyme Inhibition

**DOI:** 10.3390/molecules29235775

**Published:** 2024-12-06

**Authors:** Elżbieta Studzińska-Sroka, Magdalena Paczkowska-Walendowska, Justyna Kledzik, Agnieszka Galanty, Anna Gościniak, Piotr Szulc, Katarzyna Korybalska, Judyta Cielecka-Piontek

**Affiliations:** 1 Department of Pharmacognosy and Biomaterials, Poznań University of Medical Sciences, Rokietnicka 3, 60-806 Poznan, Poland; elastudzinska@ump.edu.pl (E.S.-S.); mpaczkowska@ump.edu.pl (M.P.-W.); agosciniak@ump.edu.pl (A.G.); jpiontek@ump.edu.pl (J.C.-P.); 2 Department of Pharmacognosy, Faculty of Pharmacy, Jagiellonian University Medical College, Medyczna 9, 30-688 Krakow, Poland; agnieszka.galanty@uj.edu.pl; 3 Department of Agronomy, Poznań University of Life Sciences, Dojazd 11, 60-632 Poznan, Poland; piotr.szulc@up.poznan.pl; 4 Department of Pathophysiology, Poznań University of Medical Sciences, Rokietnicka 8, 60-806 Poznan, Poland

**Keywords:** black elderberry, *Sambucus nigra*, wild elderberry, polyphenols, chlorogenic acid, antioxidant activity, α-glucosidase inhibition, α-amylase inhibition, extraction optimization, Box–Behnken model

## Abstract

Black elderberry (*Sambucus nigra* L.) flowers are rich in polyphenolic compounds, including chlorogenic acid and quercetin derivatives, which are known for their health benefits, particularly their antioxidant and antidiabetic properties. This study aimed to optimize the extraction conditions using the Box–Behnken model to maximize polyphenol yields from different elderberry flower cultivars and to evaluate their potential for antidiabetic action. The extracts were analyzed for their phytochemical content and assessed for enzyme inhibition, specifically targeting enzymes critical in carbohydrate digestion and glucose regulation. The anti-inflammatory activity was also assessed. Results indicated that the Black Beauty, Obelisk, and Haschberg cultivars demonstrated significant inhibition of α-glucosidase, with a high inhibitory potential against α-amylase enzymes for the Obelisk cultivar. Additionally, high chlorogenic acid content was strongly correlated with enzyme inhibition and antioxidant activity, suggesting its substantial role in glucose regulation. This study underscores the potential of elderberry flower extracts, particularly those rich in chlorogenic acid, as natural agents for managing blood glucose levels, warranting further exploration of their use in antidiabetic applications.

## 1. Introduction

Diabetes is a widespread and complex metabolic disorder characterized primarily by chronic hyperglycemia resulting from impaired insulin secretion, insulin resistance, or both [1]. Among its various forms, type 2 diabetes is the most prevalent, affecting millions globally and posing a major burden on public health systems due to its progressive nature and associated complications [2]. High postprandial blood glucose levels are of significant concern in type 2 diabetes management, as they contribute to oxidative stress, inflammation, and tissue damage, ultimately leading to complications such as cardiovascular disease, neuropathy, and retinopathy [3,4]. Consequently, managing blood glucose levels, particularly after meals, is crucial to type 2 diabetes treatment strategies [5].

One of the promising approaches to controlling postprandial blood glucose levels is the inhibition of key carbohydrate-digesting enzymes, α-glucosidase and α-amylase. These enzymes facilitate the breakdown of complex carbohydrates into glucose, which is subsequently absorbed into the bloodstream [6,7]. By inhibiting these enzymes, glucose absorption can be delayed, resulting in more controlled blood glucose levels after meals. While synthetic inhibitors such as acarbose have been developed and are widely used for this purpose, they often cause gastrointestinal side effects, including bloating and diarrhea, limiting their long-term use. This has fueled interest in identifying natural alternatives with fewer side effects, particularly those derived from plants known for their health-promoting properties [7,8,9].

In recent years, black elderberry (*Sambucus nigra* L., Wild elderberry) has gained significant attention for its rich polyphenolic and flavonoid content, which is associated with antioxidant, anti-inflammatory, and potential antidiabetic properties [10,11,12]. For medicinal purposes, black elderberry flowers are used to prepare infusions and extracts traditionally applied to support the body in combating upper respiratory tract infections [13]. One of their key effects is anti-inflammatory action, manifested by the inhibition of pro-inflammatory cytokine release by macrophages, which consequently dampens the inflammatory process [14]. Additionally, dried flowers exhibit diaphoretic properties, attributed to the presence of flavonoids in the raw material [13]. Infusions of *S. nigra* flowers are also used as a traditional expectorant. Furthermore, flower extracts demonstrate antibacterial activity [15].

A growing number of studies highlight novel aspects of the biological activity exhibited by various parts of this plant. Recent findings reveal the anti-cancer potential of elderberry fruits, including the prevention of cancerous mutations and the modulation of intracellular signaling, which collectively contribute to the reduced growth of malignant tumors and the formation of metastases in elderberry products [11]. Moreover, numerous reports in the literature describe the antiviral effects of elderberries, including activity against influenza and COVID-19 [16,17,18]. Research on elderberry flowers is rather limited and mainly focuses on their antibacterial and anti-inflammatory potential [19], as well as their antiviral activity, which has been assessed, among other things, in relation to SARS-CoV-2 and HSV [20,21].

Many cultivars of elderberry are known. They are cultivated for ornamental purposes but also hold significance for obtaining their parts for food purposes (e.g., the ‘Haschberg’ cultivar is primarily harvested for fruit processed into concentrates and juices) [22]. However, little is known about the biological activity of different parts obtained from cultivars other than wild elderberry. Studzińska-Sroka et al. studied alcoholic–aqueous extracts from the leaves of various cultivars, highlighting their interesting antioxidant and anti-inflammatory potential [23]. Additionally, blooming elderflower pollen, white elderflower buds, and blooming elderflowers of the Haschberg cultivar demonstrated antioxidant activity and a high rutin content [24]. Moreover, the fruit of the ‘Haschberg’ cultivar and the flowers of the ‘Sampo’ cultivar exhibited interesting bioactive properties (e.g., antioxidant activity) [25].

Traditionally used in herbal medicine, elderberry flowers and berries contain high amounts of bioactive compounds such as phenolic compounds, of which chlorogenic acid and quercetin derivatives occur in the highest concentrations. This translates into various beneficial effects, including reducing oxidative stress and modulating blood glucose levels [25,26]. Polyphenols, in particular, have been associated with the inhibition of α-glucosidase and α-amylase, making elderberry a compelling candidate for further research into its antidiabetic potential [27].

This research aims to establish an optimized extraction protocol to obtain polyphenol-rich extracts from elderberry flowers, characterize the extracts’ phytochemical composition across different elderberry cultivars, and evaluate their antidiabetic potential through enzyme inhibition assays. By systematically analyzing the relationship between the polyphenolic content and biological activity, this study offers a comprehensive assessment of elderberry flower extracts as potential natural agents for diabetes management, supporting the broader investigation of elderberry and other medicinal plants in chronic disease treatment strategies. Moreover, the differences between the phytochemical content and bioactivity between the different elderberry cultivars were revealed during the study, which may facilitate the preselection of the most potent ones for further investigation.

## 2. Results and Discussion

### 2.1. Optimization of the Extraction Process

**Figure 1:**
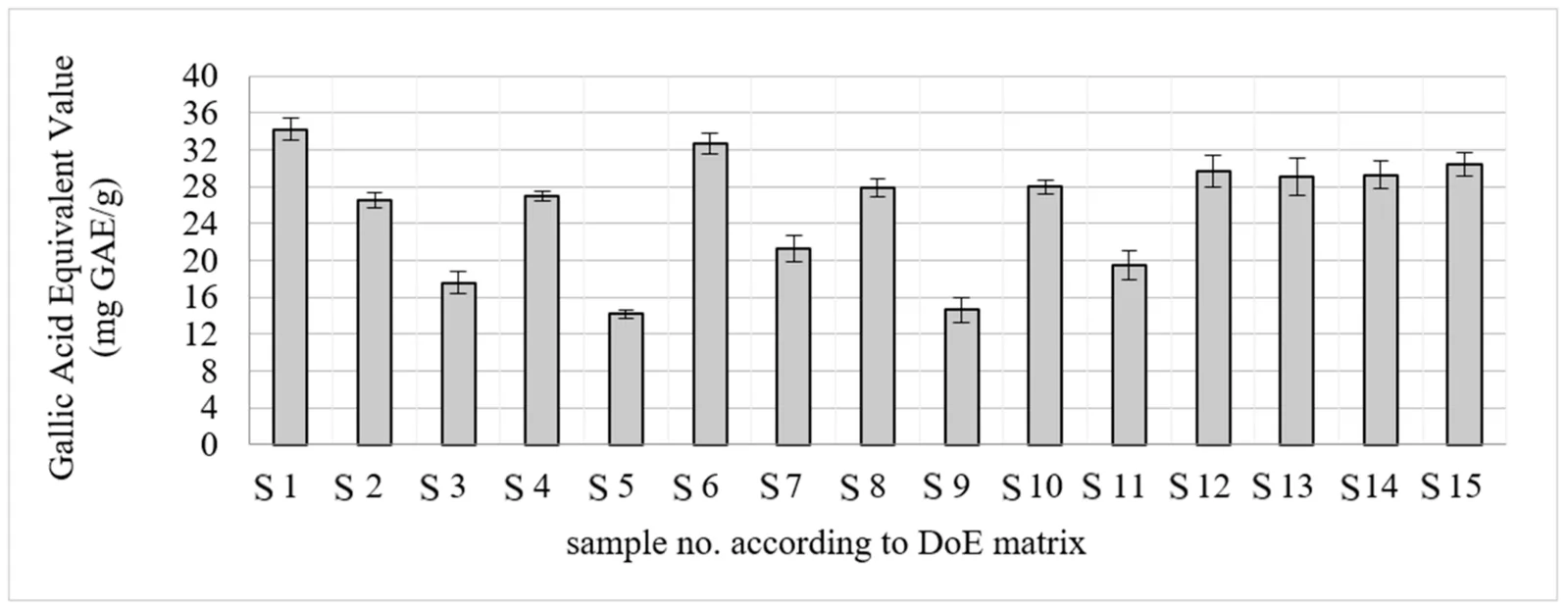
Total polyphenol content in the tested Wild elderberry samples.

Using the Box–Behnken statistical model, the extraction conditions were optimized to assess the effect of methanol content, process time, and solvent volume to plant material ratio in the extraction process of Wild elderberry flowers. The extracts were assessed for total polyphenol content using Folin–Ciocalteau reagent (Figure 1).

**Figure 2:**
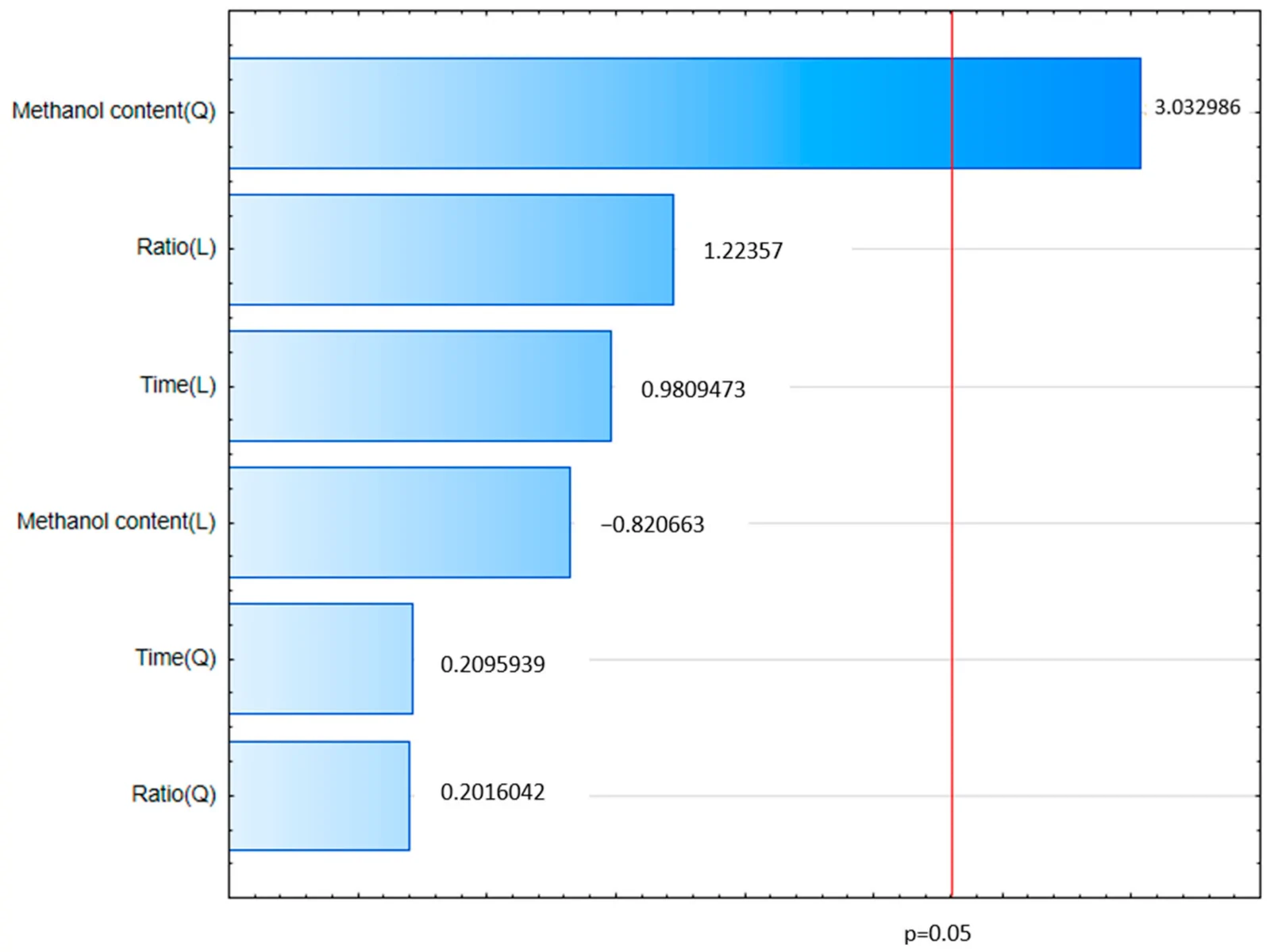
Pareto chart of standardized effects of Box–Behnken experimental analysis for total polyphenol content (TPC) in the extracts.

**Figure 3:**
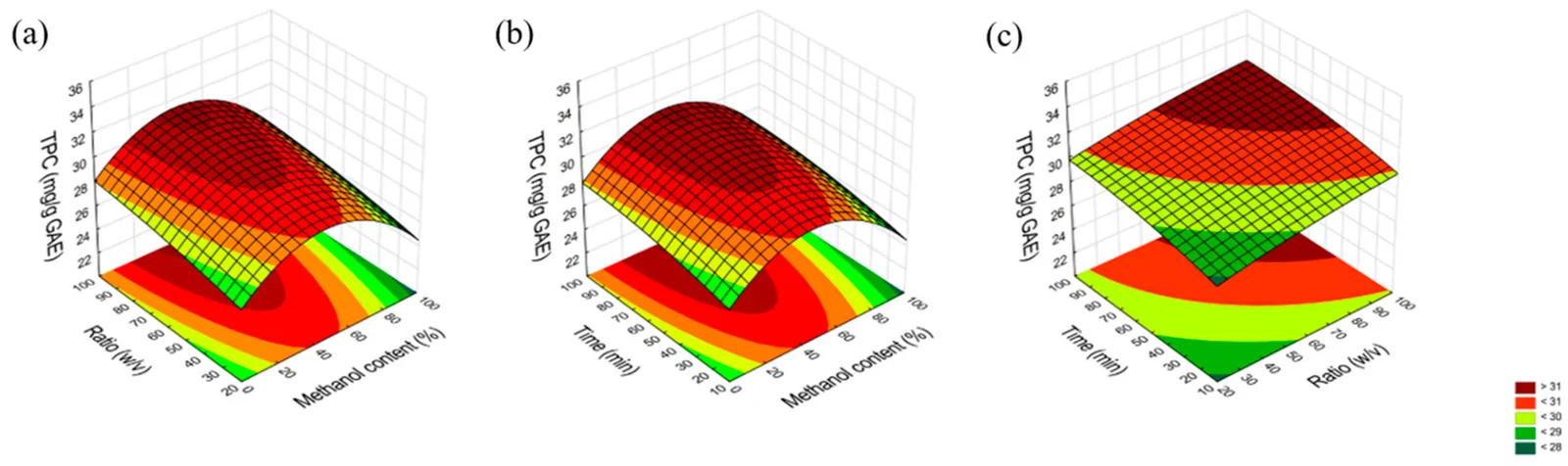
Response surface curve illustrating the effect of methanol content and plant material to solvent ratio (**a**), methanol content and time (**b**), and plant material to solvent ratio and time (**c**) on TPC.

The obtained results were entered into a statistical model to analyze the influence of the following factors: methanol content, process time, and solvent volume to sample ratio on the efficiency of the extraction of the active compounds from plant material. The analysis allowed us to determine that the optimal solvent for the extraction is 45% methanol. Factors like time and solvent volume did not significantly change the efficiency of the extraction process in relation to the adopted criterion (total polyphenol content); therefore, in order to minimize the use of time and reagents, a decision was made to adopt the lowest values of the tested process parameters (15 min, 5 × 10 mL of 45% methanol, 500 mg plant material) (Figure 2 and Figure 3).

The optimization of the extraction processes using the Box–Behnken design as a response surface methodology has been widely studied, particularly in the context of extracting bioactive compounds from various plant materials. Using this method, appropriate extraction parameters can be chosen to maximize the amount of active compounds extracted [28,29,30]. The role of methanol as a significant solvent is further supported by studies such as those by Tai et al., who optimized the extraction conditions for antioxidant compounds from banana peels, where solvent concentration was identified as a key variable [31]. Attempts have also been made to optimize the extraction of elderflowers. Oziembłowski et al. applied response surface methodology (RSM) to optimize the extraction of chlorogenic acid from elderberry flowers [32]. Their findings indicated that the extraction process could be efficiently shortened to 20 days with ethanol at a concentration of approximately 68% without significant losses in chlorogenic acid yield. Domínguez et al. demonstrated that a temperature of 60 °C, 50% ethanol concentration, and a pH of 2 were the optimal extraction parameters for chlorogenic acid [33]. The solvent composition identified in their optimization is similar to the results obtained in our study, strongly supporting the use of alcohol around 50% as an efficient solvent for polyphenol extraction from elderberry flowers. Research indicates that *Sambucus nigra* flowers possess a significant concentration of polyphenolic compounds. For instance, Viapiana et al. reported a high phenolic content ranging from 15.23 to 35.57 mg GAE/g DW for elderflowers [34]. The study by Zawiślak et al. specifically utilized the Folin–Ciocalteu method to quantify polyphenols in dried elderflowers [34]. The results indicated that the drying conditions significantly influenced the polyphenol content, suggesting that optimal processing methods are crucial for maximizing the health benefits of elderflower extracts.

### 2.2. Phytochemical Studies of the Extracts

High-performance liquid chromatography analysis allowed us to determine the content of chlorogenic acid, rutin, and isoquercetin, i.e., the active compounds in water–methanol extracts of black elderberry flowers. The highest content of chlorogenic acid was characterized by extracts from the Black Beauty (23.73 ± 0.01 mg/g of plant material) and Obelisk (21.03 ± 0.00 mg/g of plant material) cultivars (Table 1), while the lowest content was in Bez koralowy (9.39 mg/g of plant material). Cultivars rich in rutin are Haschberg (38.37 ± 0.01 mg/g of plant material) and Obelisk (30.12 ± 0.18 mg/g of plant material), while the Golden Hybrid cultivar (4.01 mg/g of plant material) is poor in this compound. Among the compounds tested, the lowest content of isoquercetin was found, regardless of the cultivar tested. The highest concentration of this flavonoid was noted in the extracts from Sambo flowers (4.69 ± 0.00 mg/g of plant material), the lowest in the Haschberg cultivar (0.09 ± 0.13 mg/g of plant material). So, it can be stated that there are significant differences in the amount of these secondary metabolites depending on the *S. nigra* cultivar studied.

Analyses of the total content of polyphenols and flavonoids were performed to deepen the phytochemical studies (Table 1 and Figure S1, Supplementary Materials). The results of these studies allowed for a better understanding of the chemical differences between the flowers of the tested *S. nigra* cultivars. In addition, the obtained data allowed for the conclusion about the potential relationship between the content of polyphenols and flavonoids in the tested raw materials and the biological activity of the tested extracts. The results of the total polyphenol analysis largely matched the results of the HPLC analysis. The Black Beauty cultivar, characterized by the highest content of chlorogenic acid, had the highest total polyphenol content (44.97 mg GAE/g). Moreover, the Black Tower (40.48 mg GAE/g) and Sambo (38.47 mg GAE/g) cultivars were particularly rich in polyphenols. It is worth noting that both the Black Beauty and Black Tower cultivars are characterized by purple-red coloration of leaves and flowers, which suggests the presence of other polyphenolic compounds from the anthocyanin group in different parts of this plant [35]. The results of the conducted evaluation of the total flavonoid content were also consistent with the results of the HPLC analysis. The highest number of flavonoids was recorded for the flowers of the Haschberg cultivar richest in rutin (18.19 mg QE/g) and the flowers of the Obelisk cultivar (16.56 mg QE/g), which was characterized by the second highest rutin content among the extracts tested. The lowest total flavonoid content was recorded for the flowers of the Sampo cultivar (9.58 mg QE/g). Interestingly, the analysis of the content of active compounds in the Wild elderberry flowers conducted simultaneously indicated that, in comparison to the other elderberry flower cultivars studied, it showed an average content of chlorogenic acid (15.39 ± 0.10), rutin (13.33 ± 0.15), and isoquercetin (1.76 ± 0.09). The extract from *S. nigra* flowers also showed a lower total content of polyphenols and flavonoids than the other cultivars studied.

Analysis of data from the literature showed that studies comparing the chemical composition of extracts from flowers of different cultivars of black elderberry are scarce and do not concern those examined by us. The assessment of the content of the active compounds in the Wild elderberry flowers was conducted earlier. It indicated that chlorogenic acid, rutin, and isoquercetin are present in Wild elderberry (*Sambuci flos*) [36]. Such analyses were conducted, among others, for alcoholic extracts, where chlorogenic acid was one of the dominant phenolic acids (318.6 ± 4.1 mg/100 g ex), rutin was the dominant flavonoid (573.5 ± 5.0 mg/100 g ex), and the content of isoquercetin was significantly lower (57.5 ± 2.7 mg/100 g ex) [37]. In turn, Viapiana and Wesołowski [10] determined the content of chlorogenic acid and rutin for infusions prepared from commercially available plant material—black elderberry flowers (6.26 mg/g of chlorogenic acid and 13.3 mg/g of rutin). Based on the obtained results and chromatograms presented in the paper, it can be stated that both chlorogenic acid and rutin are important components of most black elderberry flowers, regardless of the cultivar tested. In addition, Viapiana and Wesołowski [10] determined the total polyphenol content (TPC) for the infusions prepared from Wild elderberry (*Sambuci flos*). The determined TPC value ranged from 15.23 to 35.57 mg GAE/g of plant material. The results obtained in the present paper were slightly higher and varied depending on the cultivar tested (the lowest of the obtained values was 25.05 mg GAE/g of plant material, and the highest was 44.97 mg GAE/g). In the same experiment, the total flavonoid content in the infusions was determined. This value was also lower (from 5.27 to 13.19 mg) than the highest achieved in the present work (18.19 mg QE/g for the Haschberg cultivar). Considering the results of our research and those cited, it should be noted that the optimization of the extraction process used could have resulted in a higher content of total polyphenols and flavonoids, although the tested elderberry cultivar was also significant. In addition, regarding the lower quantities of phenolic compounds detected in Wild elderberry samples, it can be pointed out the interesting potential of other cultivars of black elderberry than those used in medicine.

### 2.3. Studies of Biological Activity of Extracts

Comparative studies of the biological activity of the extracts from twelve cultivars of black elderberry flowers were conducted for the first time. For this purpose, antioxidant activity, the ability to reduce copper ions, the ability to inhibit the enzymes α-glucosidase and α-amylase, as well as the anti-inflammatory potential were examined.

#### 2.3.1. Extracts from the Flowers of Cultivars of Black Elderberry Can Protect Against Free Radicals

Antioxidant activity plays a key role in the prevention of lifestyle diseases such as cardiovascular diseases, cancers, and neurodegenerative diseases, including diabetes control [38]. In diabetes, excess reactive oxygen species (ROS) resulting from hyperglycemia contributes to oxidative stress, which damages pancreatic cells, reducing insulin secretion and increasing insulin resistance [39]. Antioxidant activity helps to neutralize ROS, limiting cell and tissue damage and inhibiting inflammatory processes that worsen diabetic complications. Thus, the detection of biological activity studies began with the assessment of antioxidant activity. For this purpose, the ability of antioxidant substances to reduce the DPPH radical was evaluated, and the ability to reduce copper ions was assessed using the CUPRAC method (Table 2). In the study using the DPPH radical, the highest antioxidant activity was demonstrated by the Black Beauty (53.15 mg TE/g) and Obelisk (49.92 mg TE/g) cultivars. Slightly lower activity in this direction was demonstrated by Haschberg flowers (45.01 mg TE/g). These results are consistent with the increased content of the active compounds in the extracts tested. The Black Beauty cultivar also showed a superior ability to reduce copper ions (77.19 mg TE/g), suggesting that the high chlorogenic acid content increases the antioxidant properties of the raw material, while the low content determined poor antioxidant activity (Bez koralowy: 19.23 mg TE/g and 42.32 mg TE/g, for DPPH and CUPRAC, respectively). The strong antioxidant activity of chlorogenic acid was also confirmed by the standard study (IC_50_ 0.150 mg/mL). Rutin, even though it works similarly to chlorogenic acid, had a moderate effect on the activity of examined extracts.

#### 2.3.2. Extracts from the Flowers of Cultivars of Black Elderberry Inhibit α-Glucosidase More Strongly than α-Amylase

Inhibiting the enzymes α-glucosidase and α-amylase is an important mechanism in managing blood sugar levels, especially in people with type 2 diabetes [40]. A-amylase breaks down starch into smaller molecules [6], which are then digested by α-glucosidase into glucose that is absorbed into the blood [41]. Blocking these enzymes slows down the process of breaking down carbohydrates, leading to a smaller and slower rise in blood glucose after a meal. Therefore, the aim of the in vitro studies using α-glucosidase and α-amylase enzymes was to determine the hypoglycemic potential of the tested extracts from flowers of different elderberry cultivars. In the case of the inhibition of α-glucosidase, the flowers of the Black Beauty cultivar showed twice the capacity in this respect than the reference substance (acarbose) at the same concentration (55.89% and 27.85%, respectively) (Table 3). The next cultivars with high activity were Black Tower (51.39%), Samyl 1 (50.96%), and Obelisk (49.61%). The results of the inhibition of α-glucosidase are consistent with the high content of chlorogenic acid in the active extracts, which has confirmed efficacy in inhibiting this enzyme [42]. In addition, there are results in the literature suggesting that the extracts from black elderberry flowers inhibited α-glucosidase activity, with this activity being higher for alcoholic than aqueous extracts [43], while the extracts from black elderberry fruits did not inhibit α-glucosidase [44]. This suggests that the hydroalcoholic extracts from the flowers of different cultivars of black elderberry analyzed in our study may be material for further research in terms of their antidiabetic potential, which is perhaps more interesting than the anthocyanin-rich fruits of this plant.

In the case of amylase inhibition studies, most of the extracts were found to be ineffective despite the doubled concentration of the extracts. The highest capacity at a concentration of 0.1 g/mL was demonstrated by the flowers of the Obelisk cultivar (33.95%). This may be due to the high content of chlorogenic acid (21.03 mg/g of plant material) and rutin (30.12 mg/g of plant material), of which their ability to inhibit amylase is confirmed in the literature [45,46].

#### 2.3.3. Extracts from the Flowers of Black Beauty and Black Tower Cultivars Reveal the Higher Anti-Inflammatory Potential

**Figure 4:**
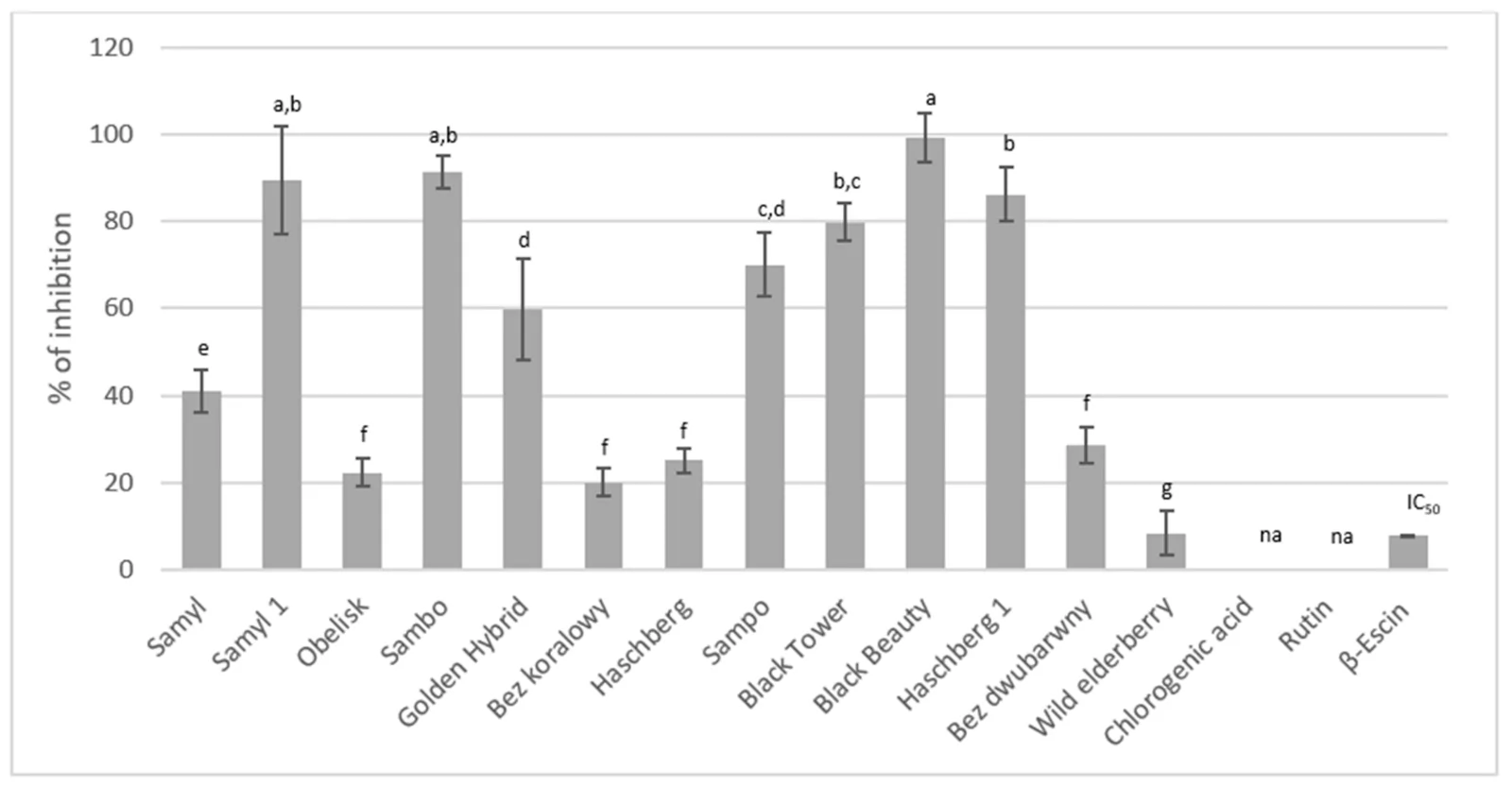
Results of the hyaluronidase inhibition test (na—not active at 25 mg/mL). Mean values with the same letter are not significantly different at *p* = 0.05 using Duncan’s test.

Chronic inflammation is a key factor in the development of many diseases. It can worsen complications such as neuropathy and retinopathy and impair wound healing [47,48]. Hyaluronidase is an enzyme that degrades hyaluronic acid, a crucial component of the extracellular matrix that promotes its breakdown, thereby weakening the protective barrier function of the skin [49]. Excessive enzymes can also generate low-molecular-weight HA fragments, which may further intensify inflammation [50]. Studies investigating hyaluronidase inhibition by the extracts from the flowers of different elderberry cultivars demonstrated varying enzyme inhibition capacities. The highest inhibition rate (99.11 ± 5.64%) was observed for the extract from the “Black Beauty” cultivar, which also contained the highest polyphenol levels (Figure 4). Significant inhibitory activity (>80%) was also shown by the “Sambo”, “Samyl”, and “Haeschberg 1” cultivars, which are all rich in isoquercetin, suggesting a substantial role for this compound in inhibiting hyaluronidase. The findings of Kim et al. confirm that isoquercetin exhibits strong inhibitory effects on this enzyme, supporting the conclusions of this study [51]. Conversely, although rutin and chlorogenic acid were dominant in the samples, they had minimal impact on inhibition. Studies have demonstrated that neither rutin nor chlorogenic acid at 25 mg/mL concentrations inhibited the enzyme, which aligns with Zhou et al., who observed weak inhibitory activity for chlorogenic acid, and with the findings of Je-Hyuk Lee, who found no inhibitory effect from rutin at low doses [52].

**Figure 5:**
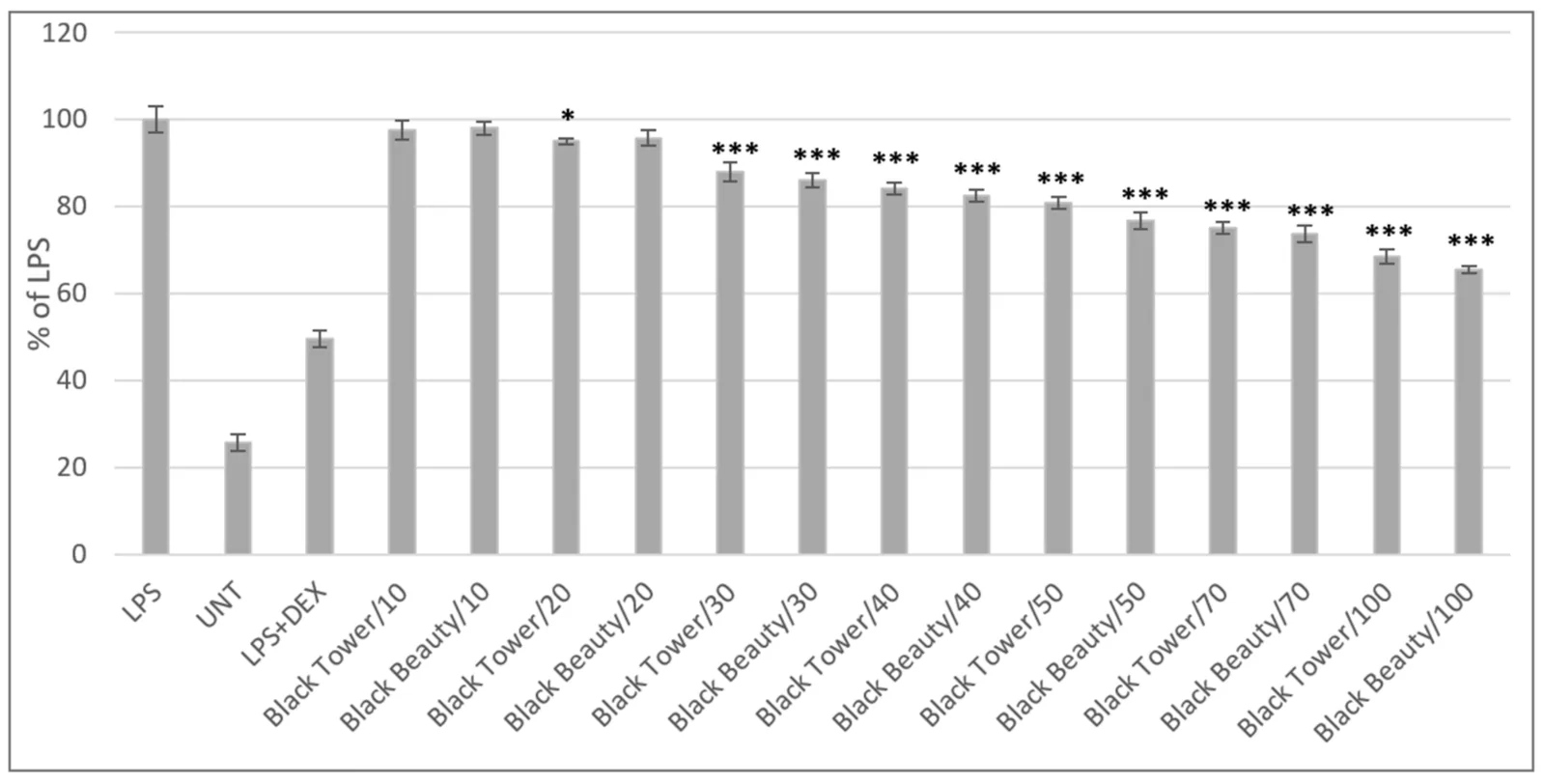
The effect of flower extracts from Black Tower and Black Beauty cultivar of elderberry on nitric oxide release in LPS-stimulated RAW 264.7 macrophages; differences statistically significant vs. LPS-treated cells, * *p* < 0.05; *** *p* < 0.001.

During the inflammation, large amounts of nitric oxide (NO) are produced, the presence of which is associated with the functioning of factors such as cytokines or bacteria. Therefore, the compounds that inhibit NO production may be inflammatory modulators. Similarly, exposure of macrophages to lipopolysaccharides (LPS) generates an inflammatory-like response [53]. For these reasons, in the next part of the experiment, the anti-inflammatory potential of the tested extracts was evaluated using the LPS-stimulated RAW 267.4 murine macrophage model, with nitric oxide as a determined indicator of an inflammatory state. First, the impact of the tested samples on macrophage viability was examined, and the results indicated that none of the tested extracts was toxic to the cells in the tested concentration range. In the highest tested concentration (100 μg/mL), cell viability did not differ significantly from the controlled, untreated cells. Thus, for the anti-inflammatory assay, the whole tested concentration range was chosen. Although most of the studied extracts did not reveal anti-inflammatory activity, samples from Black Tower and Black Beauty cultivars were the most potent in terms of the inhibition of nitric oxide release (Figure 5), and the observed effect was dose dependent. No significant differences were observed between both active extracts. However, the inhibition of NO release by the two extracts was significantly higher when compared to LPS-stimulated cells, except for the lower tested concentrations.

**Figure 6:**
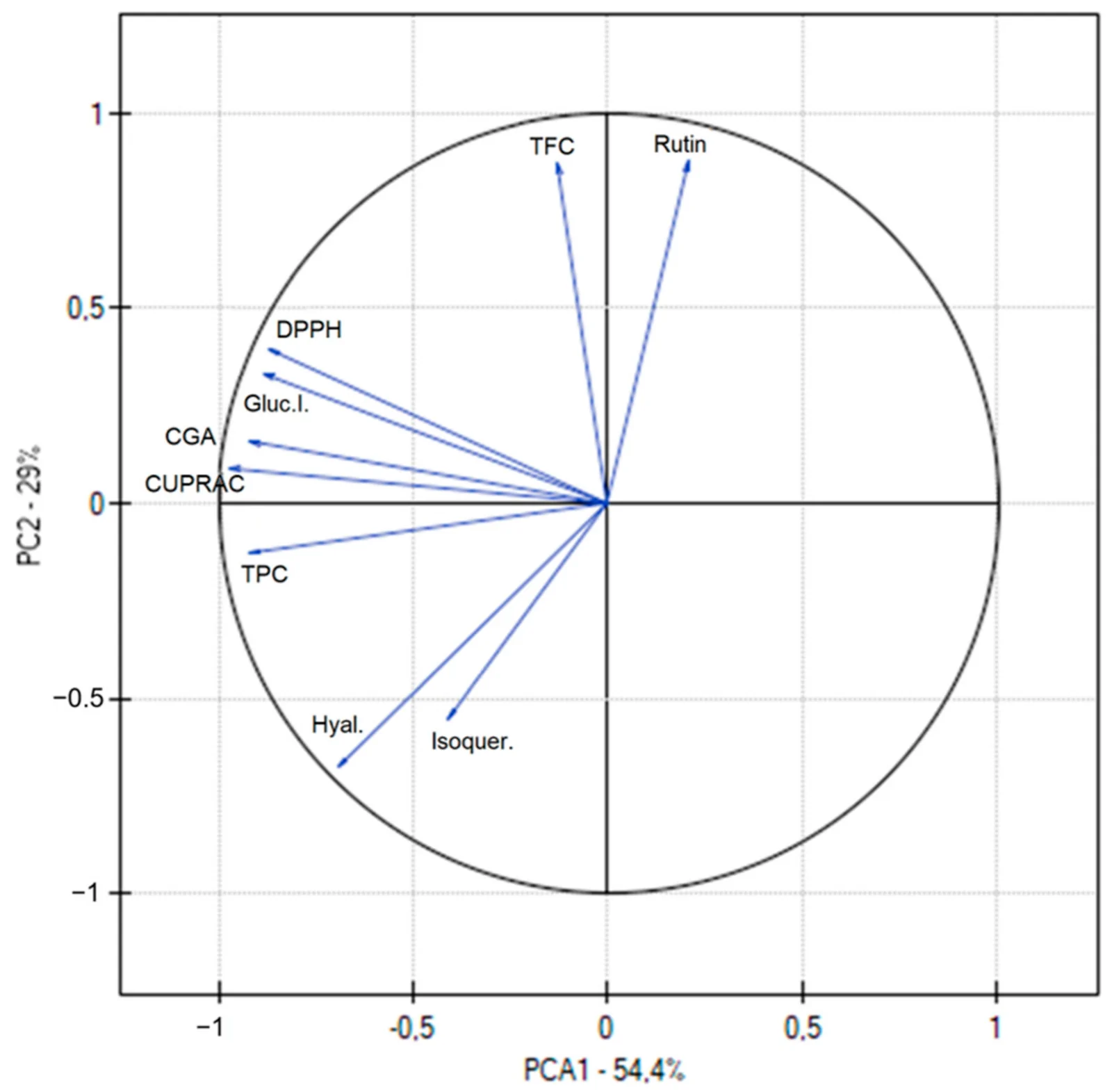
Principal component analysis (PCA) showing the factor loading plot considering active component contents (TPC, TFC, CGA, Rutin, Isoquer), antioxidant (DPPH, CUPRAC), anti-inflammatory (Hyal), and other (Gluc.I.) activities.

To analyze the relationship between the effects, Principal Component Analysis (PCA) was performed, which is a popular data analysis method that allows observing regularities between the studied variables. Figure 6 shows the results of PCA of extracts, considering all cultivars. A strong positive correlation was observed between chlorogenic acid content, TPC and DPPH, chlorogenic acid content, TPC and CUPRAC, as well as between rutin and TFC, which was described previously. Moreover, a strong correlation was noticed between chlorogenic acid content and α-glucosidase inhibition. The above indicates an important role of chlorogenic acid content in the activity of the plant material. It was also noted that there was a strong positive correlation between TPC and hyaluronidase inhibition, which indicated an important role of polyphenol components in biological activity (Table S1, Supplementary Materials).

## 3. Materials and Methods

### 3.1. Chemical Reagents

Sodium carbonate, sodium hydroxide, DMSO, formic acid, methanol, ammonium acetate, and copper (II) chloride were purchased from Avantor Performance Materials Poland S.A. (Gliwice, Poland). HPLC-grade acetonitrile and water, as well as Folin–Ciocalteu phenol reagent, were from Merck (Darmstadt, Germany). Acarbose, β-escin, chlorogenic acid, gallic acid, isoquercetin, quercetin, rutin, and Trolox were purchased from Sigma Aldrich (St. Louis, MO, USA). All other chemicals were from the Sigma–Aldrich Chemical Co. (St. Louis, MO, USA). High-quality pure water and ultra-high-quality pure water were prepared using a Direct-Q 3 UV Merck Millipore purification system (Merck, Darmstadt, Germany).

### 3.2. Plant Material

The plant material tested was dried flowers of black elderberry cultivars: (1) Samyl, (2) Samyl 1, (3) Obelisk, (4) Sambo, (5) Golden Hydrid, (6) Bez koralowy, (7) Haschberg, (8) Sampo, (9) Black Tower, (10) Black Beauty, (11) Haschberg 1, and (12) Bez dwubarwny. The black elderberry cultivar flowers were air-dried in a dry, ventilated place. The Wild elderberry flowers were purchased and dried from a Polish herb packaging herbal company “KAWON—HURT” (Gostyń, Poland).

The plant material for the tests came from the experimental station area of the Research Centre for Cultivar Testing (COBORU) in Słupia Wielka. The material was provided for the tests thanks to the kindness of Prof. Piotr Szulc from the Department of Agronomy of the University of Life Sciences in Poznań [23].

**Figure 7:**
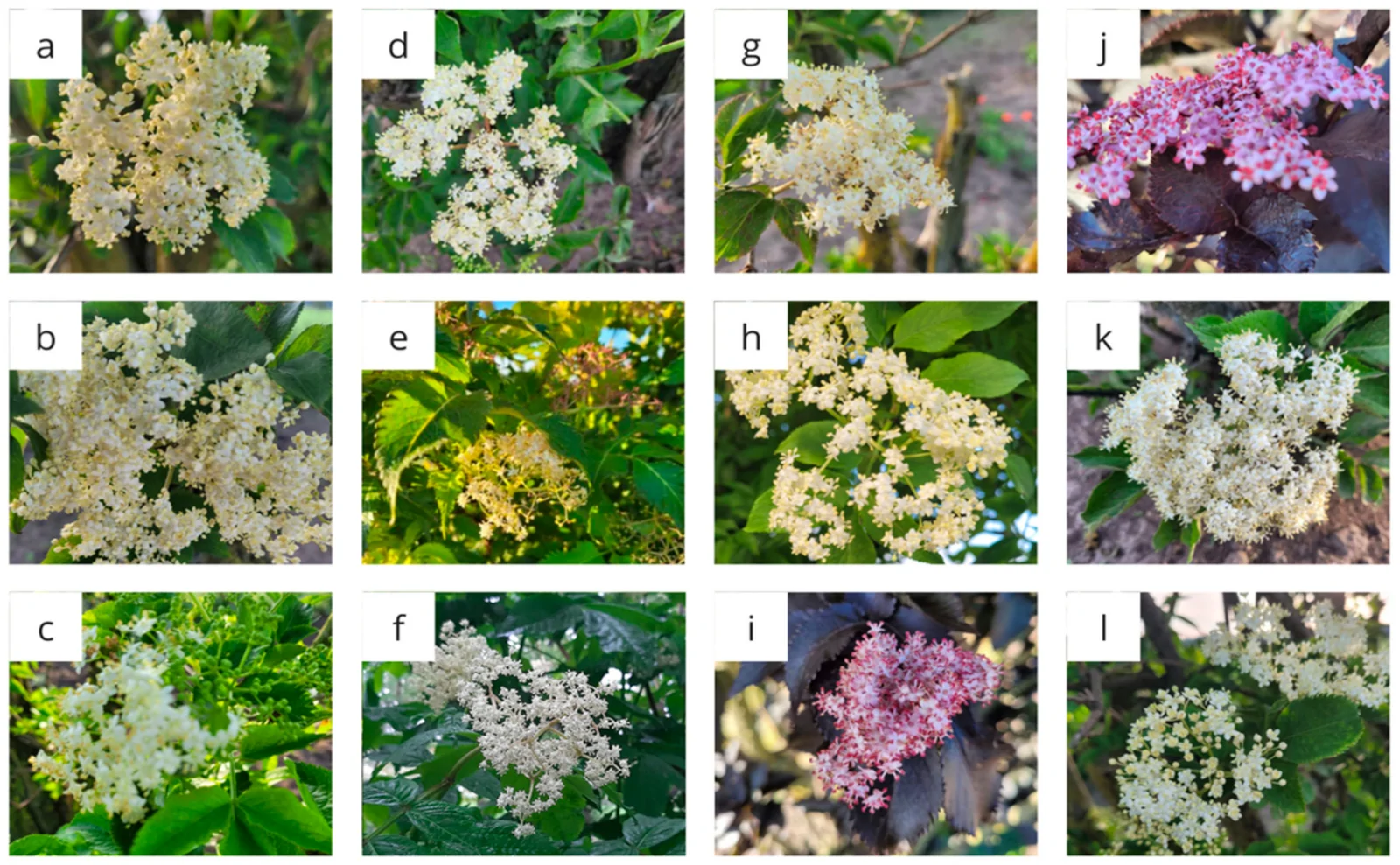
Flowers of black elderberry cultivars: Samyl (**a**), Samyl 1 (**b**), Obelisk (**c**), Sambo (**d**), Golden Hybrid (**e**), Bez koralowy (**f**), Haschberg (**g**), Sampo (**h**), Black Tower (**i**), Black Beauty (**j**), Haschberg 1 (**k**), Bez dwubarwny (**l**).

The appearance of the flowers of the tested cultivars is shown in Figure 7.

### 3.3. Optimization of the Extraction Process

The Box–Behnken statistical design was used to develop the model and to investigate and evaluate the influence of input factors on the experimental result using Statistica 13.3 software (TIBCO Software Inc., Palo Alto, CA, USA). The input data for Wild elderberry extraction were the influence of three variables—the influence of process time, methanol concentration, and the ratio of plant material mass to solvent (expressed in the experimental plan as the volume of solvent used). The output data were the content of polyphenols in the extracts assessed by the Folin–Ciocalteu method. The following constant parameters were assumed: (1) plant material weight: 500 mg, (2) extraction temperature: 50 °C, and (3) number of extraction repetitions: 5.

Taking these basics into account, the experimental plan was generated and placed in Table 4. Fifteen extracts (S1–S15) were obtained.

The experiments were performed on the Wild elderberry flowers. The weighed portions, together with the solvent, were placed in falcons sealed with parafilm. The extraction was performed in an ultrasonic bath. Then, the extracts were filtered through cotton wool into a beaker, in which the filtrate from each repetition stage was combined. Due to the threefold repetition of the extraction, the following sample volumes were obtained: 30 mL, 60 mL, and 150 mL.

In the next stage, the volume of the extracts was equalized to 100 mL. For this purpose, the 30 mL and 60 mL samples were supplemented with the appropriate solvent concentration for the given sample. The obtained 150 mL samples were concentrated using an evaporator to a volume of 90 mL and then supplemented with the appropriate solvent.

In order to test the content of polyphenols in the obtained extracts, 1 mL samples were taken into 2 mL Eppendorf bottles. Samples were evaporated and then dissolved in 1 mL of DMSO, which prevented precipitation during analysis.

#### 3.3.1. Determination of Total Polyphenol Content (TPC) Using the Folin–Ciocalteu Reagent

The analysis was performed following the method described by Studzińska-Sroka et al. [23]. In short, 25.0 µL of the tested extract or standard, 200.0 µL of distilled water, 15.0 µL of Folin–Ciocalteu reagent, and 60.0 µL of a 20% calcium carbonate solution were added sequentially to the wells. A blank sample, consisting of the reagents without the tested extract or reference compound, was used as a control. The plate was shaken for 5 min at 600 rpm (at room temperature and in the dark), followed by a 25 min incubation. The absorbance was measured at a wavelength of 760 nm using a microplate reader (Multiskan GOx1510 from Thermo-Scientific, Waltham, MA, USA). The results were the average from *n* = 6 measurements and were expressed as milligrams of gallic acid equivalent (GAE)/g dry plant material ± standard deviation (SD).

### 3.4. Extraction and Preparation of Extracts for Testing

Due to the results of the extraction process optimization, the following extraction conditions were adopted for further studies:

(1) Plant material weight: 500 mg;
(2) Extraction temperature: 50 °C;
(3) Methanol concentration: 45%;
(4) Process time: 15 min;
(5) Solvent volume: 10 mL;
(6) Number of extraction repetitions: 5.

The weights, together with the solvent, were placed in conical flasks and extracted in an ultrasonic bath. The extracts were then filtered through cotton wool, and the filtrate from each repetition was collected in round-bottom flasks. The extracts obtained were concentrated on an evaporator to a volume of approx. 10 mL, transferred to 10 mL measuring flasks, made up to commensurability using a 45% methanol solution, and then divided into 6 Eppendorf flasks. The extracts were frozen at −20 °C and stored for further studies.

### 3.5. Phytochemical Studies of the Extracts

#### 3.5.1. Chemical Analysis of the Extract Using High-Performance Liquid Chromatography (HPLC)

Using high-performance liquid chromatography, the presence and content of chlorogenic acid, rutin, and isoquercetin were detected and determined in extracts from flowers of different cultivars of black elderberry. The analysis was carried out using the method described by Paczkowska-Walendowska et al. [54]. To ensure the reliability of the obtained results, the method was validated. Process parameters: detection wavelength: 325 nm (chlorogenic acid), 360 nm (rutin, isoquercetin); mobile phase flow: 1 mL/min; column temperature: 40 °C; mobile phase: A- 0.1% formic acid, B-acetonitrile, according to the gradient: 0–35 min: 2–20% B; 35–55 min: 20–70% B; 55 min: 2% B; 55–60 min: 2% B.

Analysis procedure: Solutions of standard substances were prepared by weighing and dissolving aliquots in HPLC-grade methanol (chlorogenic acid 1 mg/mL and 0.1 mg/mL, rutin 0.5 mg/mL, isoquercetin 0.5 mg/mL). The obtained solutions were filtered through a membrane filter (0.45 µL). The following injection volumes were made: chlorogenic acid 1 mg/mL: 12, 10, 8, 6, 4, 2, 1, and 0.1 mg/mL: 8, 6, 4 µL; rutin 0.5 mg/mL: 80, 60, 40, 20, 10, 5 µL; isoquercetin 0.5 mg/mL: 12, 8, 6, 4, 2, 1 µL. The standard curve was determined, and the method was validated by determining the parameters, i.e., linearity, indirect and direct precision, limit of quantification (LOQ), and limit of detection (LOD).

#### 3.5.2. Determination of Total Polyphenol Content (TPC)

Total polyphenol content was determined according to the procedure described in Section 3.3.1, except the extracts were prepared at a concentration of 1.5625 mg/mL.

#### 3.5.3. Determination of Total Flavonoid Content (TFC) Using Aluminum Chloride

The analysis was performed following the method described by Studzińska-Sroka et al. [55]. In short, 100.0 µL of the tested extract or standard was mixed with 100.0 µL of 2% methanolic solution of aluminum chloride. The absorbance was read at 415 nm using the microplate reader (Multiskan GO 1510, Thermo Fisher Scientific, Vantaa, Finland). The blank contained extract and methanol instead of a 2% methanolic solution of aluminum chloride. The results were the average from *n* = 6 measurements and were expressed as milligrams of quercetin equivalent (QE)/g dry plant material ± standard deviation (SD).

### 3.6. Studies of Biological Activity of Extracts

#### 3.6.1. Antioxidant Activity Assay

The antioxidant activity of the extracts was determined as the ability to reduce the activity of the DPPH radical and the ability to reduce copper ions using the CUPRAC method. Results are expressed in Trolox equivalent (TE mg/g).

##### DPPH Assay

A previously described DPPH analysis was used to determine the antiradical activity [23]. Briefly, 25.0 μL of the tested extract, prepared in concentrations of 0.78125 mg of dry plant material per mL, was combined with 175.0 μL of DPPH solution (3.9 mg/50 mL of methanol). The mixture was shaken in the dark at 500 rpm for 30 min at room temperature. Absorbance was measured at 517 nm using a plate reader (Multiskan GO 1510, Thermo Fisher Scientific, Vantaa, Finland). Each test was conducted in duplicate, and the final result was calculated as the average of six measurements (*n* = 6). Trolox, at concentrations ranging from 0.125 to 0.0078125 mg/mL, served as the positive control.

##### CUPRAC Assay

The CUPRAC analysis was conducted using the procedure described previously [23]. The CUPRAC reagent was prepared by mixing equal volumes of neocuproine solution (7.5 mM), copper (II), chloride solution (10 mM), and ammonium acetate buffer (1 M, pH = 7.0). Then, 50 μL of the tested extract (1.5625 mg of dry plant material per mL) and 150 μL of the CUPRAC reagent were mixed. The absorbance was measured after a 30 min incubation in a dark place and at room temperature, at a wavelength of λ = 450 nm. The blank was an attempt to replace 50.0 μL of the tested extract with 50.0 μL of extraction solvent. The assay was repeated twice, and the result represent the average of seven determinations (*n* = 7). Trolox, at concentrations ranging from 0.2 to 0.00625 mg/mL, served as the positive control.

#### 3.6.2. α-Glucosidase Inhibition Assay

The inhibition of α-glucosidase by the extracts was conducted based on the method by Studzińska-Sroka et al. [55], with some modifications. In brief, 50.0 μL of the sample solution (3.125 mg dry plant material/mL) or positive control (acarbose at 0.78125–25 mg/mL) were pre-incubated with 50.0 μL of 0.1 M phosphate buffer (pH 6.8) and 30.0 µL of α-glucosidase solution (0.5 U/mL) in 96-well plates at 37 °C for 15 min. Then, 20.0 μL of 5 mM p-nitrophenyl-α-D-glucopyranoside (pNPG) in 0.1 M phosphate buffer was added and incubated at 37 °C for 20 min. The reaction was stopped by adding 100.0 µL of 0.2 M sodium carbonate, and the absorbance was measured at 405 nm after 2 min using the microplate reader (Multiskan GO 1510, Thermo Fisher Scientific, Vantaa, Finland). The control absorbance was measured without extracts/acarbose, and the absorbance of extract/compound solution without enzyme was used as the blank for the tested sample. The results were the average from *n* = 7 measurements. The enzyme inhibition rate was expressed as a percentage of inhibition based on the final concentration of the substance in the enzymatic reaction.

#### 3.6.3. α-Amylase Inhibition Assay

The inhibition of α-glucosidase by the extracts was conducted based on the method by Studzińska-Sroka et al. [55], with some modifications. In brief, 50.0 μL of the sample solution (100 mg dry plant material/mL) or positive control (acarbose at 0.005–1.25 mg/mL) were preincubated with 20 µL of α-amylase solution prepared by dissolving in phosphate buffer with pH = 6.9 (2.0 U/mL) at 37 °C for 20 min. Then, 20.0 μL of amylase solution (0.5% in the buffer) was added, and the plate was incubated again at 37 °C for 20 min. Then, 60 µL of color reagent (96 mM 3,5-dinitrosalicylic acid solution, 5.31 M potassium sodium tartrate solution in 2 M sodium hydroxide, and deionized water) was added, and the plate was placed in an oven heated to 90 °C. After 45 min, 100 µL of distilled water was added to the samples, and their absorbance was measured at a wavelength of λ = 540 nm using the microplate reader (Multiskan GO 1510, Thermo Fisher Scientific, Vantaa, Finland). The control sample consisted of a mixture of 20 µL of distilled water and 20 µL of amylase solution, while the blank consisted of a mixture of 20 µL of distilled water and 20 µL of phosphate buffer. The results were the average from *n* = 6 measurements. The enzyme inhibition rate was expressed as a percentage of inhibition based on the final concentration of the substance in the enzymatic reaction.

#### 3.6.4. Anti-Inflammatory Activity Assay

##### Hyaluronidase Inhibition Assay

The anti-hyaluronidase activity was performed according to the previously described method [23]. Briefly, 25.0 µL of incubation buffer, 25.0 µL of enzyme solution (30 U/mL), 10.0 µL of the tested extract (250 mg/mL), and 15.0 µL of acetate buffer were mixed in the well. After 15 min of incubation (37 °C) with shaking, 25.0 µL of hyaluronic acid (HA) solution was added and incubated for 45 min with shaking (37 °C). After this time, 200.0 µL of CTAB solution in 2% sodium hydroxide was added. After 10 min of incubation without shaking (at room temperature), the absorbance (λ = 600 nm) was measured using a plate reader (Multiskan GO 1510, Thermo Fisher Scientific, Vantaa, Finland). The composition of blanks was previously described [43]. The determination was repeated twice and calculated from four results (*n* = 4) for samples and three (*n* = 3) for reference (β-escin). The results are expressed as a percentage of inhibition ± SD. β-Escin in the concentration range of 10.0–5.0 mg/mL was used as a positive control.

##### Determination of Anti-Inflammatory Potential in RAW 264.7 Model

Murine RAW 264.7 macrophages were grown in DMEM high glucose, supplemented with 10% fetal bovine serum and 1% antibiotics solution. Before the main experiment, the cytotoxic potential of the tested samples to RAW264.7 macrophages was assessed using the MTT assay in the concentration range of 10–100 μg/mL, as described previously [56]. For the anti-inflammatory assay, the cells were seeded onto 96-well plates (1.5 × 105 cells/well) and pre-treated with the tested samples (at the concentration range indicated above) or dexamethasone (0.5 μg/mL) as a reference drug for 1 h. Cells treated with LPS alone were used as a positive control, and untreated cells were included in the experiment. Subsequently, 10 ng/mL of LPS was added to induce inflammation, as previously described [56], and the incubation was performed for 24 h. Cell culture supernatants were used for the determination of the nitric oxide level, using Griess Reagent Kit (Promega Corporation, Madison, Winooski, VT, USA), according to the manufacturer’s protocol. The results were expressed as a percentage of the LPS control.

### 3.7. Statistical Analysis

The data that were gathered was expressed using the means ± SD. The statistical methodology employed in this study was a one-way analysis of variance (ANOVA), and statistical differences were computed using Duncan’s post hoc tests or a post hoc Tukey’s test (anti-inflammatory activity) and a significance threshold of *p* < 0.05 using Statistica 13.3 software (Statsoft, Krakow, Poland). Principal component analysis (PCA) was used to analyze correlations using Statistica 13.1 and PQStat software version 1.8.4.142.

## 4. Conclusions

Our study aims to provide initial insights into the bioactive compounds and in vitro bioactivities of different elderberry cultivars flowers. The results highlight the promising antidiabetic potential of examined extracts, specifically targeting their ability to inhibit enzymes involved in carbohydrate digestion, such as α-glucosidase and α-amylase. Optimized extraction conditions, determined through the Box–Behnken model, maximized the yields of bioactive compounds, particularly polyphenols like chlorogenic acid and rutin. Among the tested cultivars, Black Beauty, Obelisk, and Haschberg exhibited the highest levels of these compounds, correlating with significant anti-diabetic enzyme inhibition and antioxidant activity. Additionally, among these cultivars, Black Beauty was the most active as an anti-inflammatory agent, as evidenced by both cell-based and cell-free tests.

The PCA findings suggest that chlorogenic acid plays a pivotal role in the antidiabetic potential of elderberry flower extracts, with high content linked to effective α-glucosidase inhibition and antioxidant activity. While most extracts displayed limited α-amylase inhibition, the potent α-glucosidase inhibition aligns with the goal of managing postprandial blood glucose levels. Additionally, the strong antioxidant activity further supports these extracts’ potential role in mitigating oxidative stress—a key factor in diabetes complications, and the anti-inflammatory potential may have a protective effect against diabetes complications.

Overall, black elderberry flower extracts, especially those from specific cultivars, show potential as natural agents for blood glucose regulation, offering a basis for further investigation into their integration into antidiabetic treatments. Further research should focus on using ethanol extracts and, above all, on conducting in vivo studies to validate these in vitro findings and explore the practical applications of elderberry flower extracts, especially of the Black Beauty cultivar in diabetes management.

## Figures and Tables

**Table 1:** Content of chlorogenic acid, rutin, isoquercetin, TPC, and TFC in flower extracts of elderberry cultivars.

Cultivar	Content [mg of Standard/g of Plant Material]			TPC [mg GAE/g]	TFC [mg QE/g]
	Chlorogenic Acid	Rutin	Isoquercetin		
Samyl	19.35 ± 0.16 ^c^	25.70 ± 0.11 ^c^	0.16 ± 0.01 ^h^	33.00 ± 0.49 ^e^	12.52 ± 0.23 ^g^
Samyl 1	19.40 ± 0.07 ^c^	14.35 ± 0.22 ^g^	2.13 ± 0.04 ^c^	36.00 ± 0.77 ^d^	13.90 ± 0.48 ^e^
Obelisk	21.03 ± 0.00 ^b^	30.12 ± 0.18 ^b^	1.91 ± 0.11 ^d^	36.48 ± 1.09 ^d^	18.65 ± 0.25 ^b^
**Sambo**	19.46 ± 0.00 ^c^	10.63 ± 0.06 ^k^	**4.69 ± 0.00** ^a^	38.47 ± 1.75 ^c^	13.44 ± 0.31 ^f^
Golden Hybrid	16.00 ± 0.02 ^g^	4.01 ± 0.01 ^l^	0.53 ± 0.02 ^g^	32.14 ± 1.38 ^e,f^	16.68 ± 0.35 ^c,d^
Bez koralowy	9.39 ± 0.05 ^j^	22.41 ± 0.27 ^d^	0.22 ± 0.01 ^h^	25.05 ± 1.12 ^h^	13.15 ± 0.14 ^f^
**Haschberg**	18.57 ± 0.44 ^d^	**38.37 ± 0.01** ^a^	0.09 ± 0.03 ^h^	30.80 ± 0.89 ^f^	**20.29 ± 0.20** ^a^
Sampo	16.78 ± 0.05 ^f^	11.33 ± 0.15 ^j^	1.78 ± 0.11 ^d,e^	32.40 ± 0.67 ^e,f^	11.68 ± 0.17 ^h^
Black Tower	17.30 ± 0.02 ^e^	16.58 ± 0.03 ^f^	1.72 ± 0.00 ^e^	40.48 ± 1.04 ^b^	16.28 ± 0.09 ^d^
**Black Beauty**	**23.73 ± 0.01** ^a^	12.75 ± 0.01 ^i^	1.10 ± 0.21 ^f^	**44.97 ± 1.15** ^a^	14.27 ± 0.18 ^e^
Haschberg 1	18.46 ± 0.06 ^d^	10.51 ± 0.00 ^k^	4.46 ± 0.05 ^b^	34.95 ± 1.23 ^d^	12.72 ± 0.12 ^g^
Bez dwubarwny	14.78 ± 0.15 ^i^	20.09 ± 0.30 ^e^	1.84 ± 0.12 ^d,e^	32.09 ± 0.61 ^e,f^	16.84 ± 0.23 ^c^
Wild elderberry	15.39 ± 0.10 ^h^	13.33 ± 0.15 ^h^	1.76 ± 0.09 ^d,e^	28.57 ± 0.77 ^g^	11.46 ± 0.23 ^h^

Mean values within a column with the same letter are not significantly different at *p* = 0.05 using Duncan’s test; the highest value is in bold.

**Table 2:** Antioxidant activity results for the tested cultivars expressed as Trolox equivalents (TE mg/g), determined by the DPPH and CUPRAC methods, and the main compound measured as references.

Cultivar	DPPH [mg TE/g]	CUPRAC [mg TE/g]
Samyl	38.30 ± 4.64 ^c,d^	55.10 ± 0.92 ^g^
Samyl 1	39.61 ± 1.80 ^c,d^	70.32 ± 0.70 ^b^
Obelisk	49.92 ± 2.40 ^a^	68.25 ± 1.00 ^c^
Sambo	40.09 ± 1.82 ^c^	67.80 ± 2.06 ^c^
Golden Hybrid	33.22 ± 1.03 ^e,f^	62.46 ± 1.73 ^e^
Bez koralowy	19.23 ± 0.64 ^h^	42.32 ± 0.99 ^j^
Haschberg	45.01 ± 1.12 ^b^	65.71 ± 0.80 ^d^
Sampo	31.57 ± 1.29 ^f,g^	59.16 ± 1.41 ^f^
Black Tower	38.18 ± 2.16 ^c,d^	67.71 ± 1.09 ^c^
**Black Beauty**	**53.15 ± 0.85** ^a^	**77.19 ± 1.21** ^a^
Haschberg 1	41.16 ± 2.41 ^c^	62.71 ± 1.00 ^e^
Bez dwubarwny	35.92 ± 2.00 ^d,e^	52.70 ± 1.11 ^h^
Wild elderberry	29.34 ± 2.47 ^g^	50.47 ± 0.21 ^i^
Chlorogenic acid	0.150 ± 0.011 IC_50_ [mg/mL]	25.70 ± 0.52 IC_0.5_ [mg/mL]
Rutin	0.148 ± 0.000 IC_50_ [mg/mL]	39.86 ± 0.21 IC_0.5_ [mg/mL]

Mean values within a column with the same letter are not significantly different at *p* = 0.05 using Duncan’s test; the highest value is in bold.

**Table 3:** Results of the α-glucosidase and α-amylase inhibition test.

Cultivar	α-Glucosidase Inhibition [%]	α-Amylase Inhibition [%]
Samyl	42.31 ± 1.67 ^e^	nd
Samyl 1	50.96 ± 1.79 ^b^	nd
Obelisk	49.61 ± 1.36 ^b,c^	33.95 ± 3.48 ^a^
Sambo	48.34 ± 1.70 ^b,c,d^	13.48 ± 1.45 ^c^
Golden Hybrid	46.72 ± 1.11 ^c,d^	22.89 ± 2.77 ^b^
Bez koralowy	34.30 ± 3.52 ^f^	nd
**Haschberg**	**55.58 ± 2.42** ^a^	nd
Sampo	45.25 ± 1.25 ^d,e^	nd
Black Tower	51.39 ± 0.83 ^b^	nd
**Black Beauty**	**55.89 ± 1.51** ^a^	nd
Haschberg 1	46.83 ± 1.51 ^c,d^	13.70 ± 2.28 ^c^
Bez dwubarwny	42.24 ± 1.51 ^e^	22.70 ± 2.34 ^b^
Wild elderberry	29.74 ± 1.21 ^g^	nt
Chlorogenic acid	0.509 ± 0.021 IC_50_ [mg/mL]	nt
Rutin	0.390 ± 0.011 IC_50_ [mg/mL]	nt
Acarbose 3.125	27.85 ± 1.70	nt
Acarbose	8.24 ± 0.55 IC_50_ [mg/mL]	0.011 ± 0.001 IC_50_ [mg/mL]

nd—no activity demonstrated; nt—not tested. Mean values within a column with the same letter are not significantly different at *p* = 0.05 using Duncan’s test; the highest value is in bold.

**Table 4:** Extraction process experiment plan.

Lp.	Methanol Content [%]	Time [min]	Solvent Volume [mL]
S1	50	15	50
S2	100	52.5	10
S3	0	52.5	50
S4	100	52.5	50
S5	0	52.5	10
S6	50	52.5	30
S7	100	15	30
S8	50	15	10
S9	0	15	30
S10	100	90	30
S11	0	90	30
S12	50	90	50
S13	50	90	10
S14	50	52.5	30
S15	50	52.5	30

## Data Availability

All data supporting reported results can be found within the manuscript and Supplementary Materials.

## Supplementary Materials

The following supporting information can be downloaded at https://www.mdpi.com/article/10.3390/molecules29235775/s1. Figure S1. Chromatograms of 45% hydroalcoholic extracts from the flowers of each tested elderberry cultivars: Samyl (a), Samyl 1 (b), Obelisk (c), Sambo (d), Golden Hybrid (e), Red Elderberry (f), Haschberg (g), Sampo (h), Black Tower (i), Black Beauty (j), Haschberg 1 (k), Bez dwubarwny (l) and Wild elderberry (m). Peaks correspond to chlorogenic acid (1), rutin (2), and isoquercitrin (3); Table S1. Correlation matrix.
