# Supplementary material for: Antidiabetic Potential of Black Elderberry Cultivars Flower Extracts: Phytochemical Profile and Enzyme Inhibition
Source: Molecules. 2024 Dec 6;29(23):5775. doi: 10.3390/molecules29235775 (PMC11643853; doi:10.3390/molecules29235775)
Supplement: Supplementary file 1 [file molecules-29-05775-s001.zip › molecules-3328459-supplementary.pdf]

Antidiabetic Potential of Black Elderberry Cultivars Flower Extracts: Phytochemical Profile and Enzyme Inhibition

Supplementary material

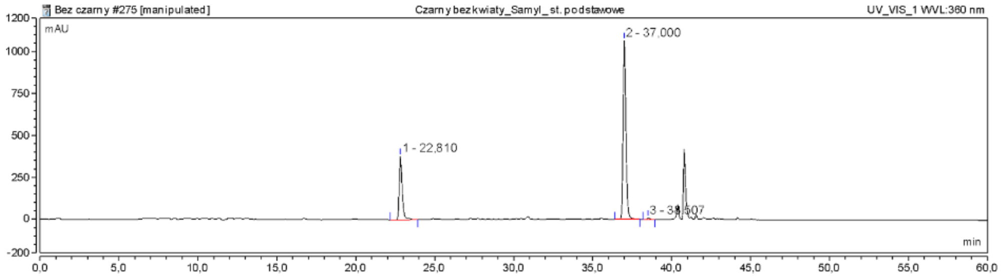
Bez czarny #275 [manipulated]
Czarny bez kwiaty_Samyl_st. podstawowe
UV_VIS_1 WVL:360 nm
mAU
1 - 22,810
2 - 37,000
3 - 38,507
min

a.

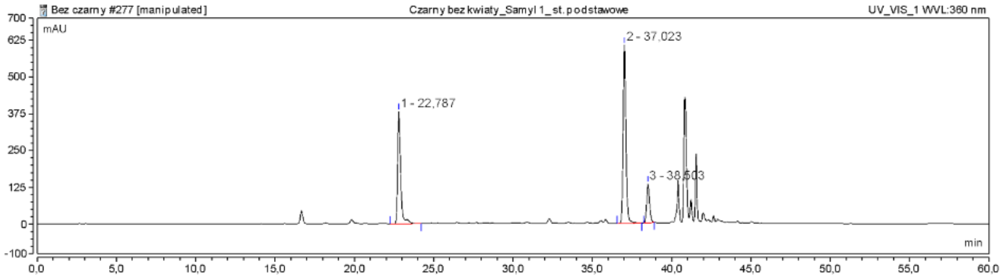
Bez czarny #277 [manipulated]
Czarny bez kwiaty_Samyl 1_st. podstawowe
UV_VIS_1 WVL:360 nm
mAU
1 - 22,787
2 - 37,023
3 - 38,503
min

b.

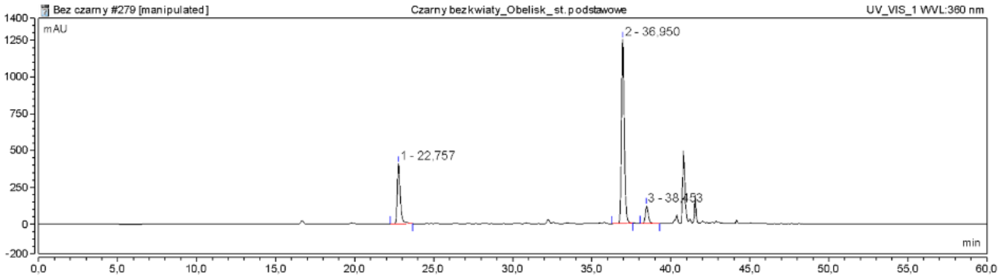
Bez czarny #279 [manipulated]
Czarny bez kwiaty_Obelisk_st. podstawowe
UV_VIS_1 WVL:360 nm
mAU
1 - 22,757
2 - 36,950
3 - 38,453
min

c.

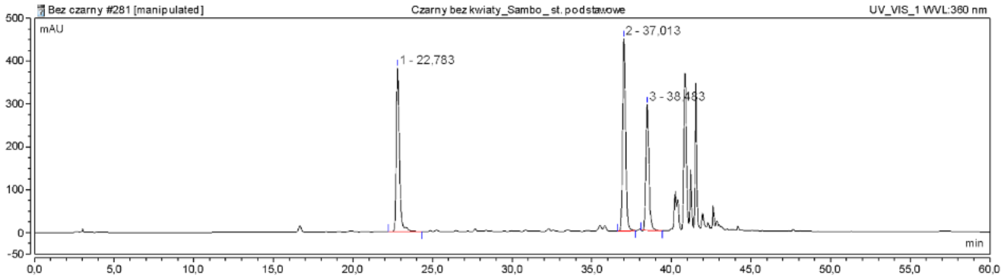
Bez czarny #281 [manipulated]
Czarny bez kwiaty_Sambo_st. podstawowe
UV_VIS_1 WVL:360 nm
mAU
1 - 22,783
2 - 37,013
3 - 38,483
min

d.

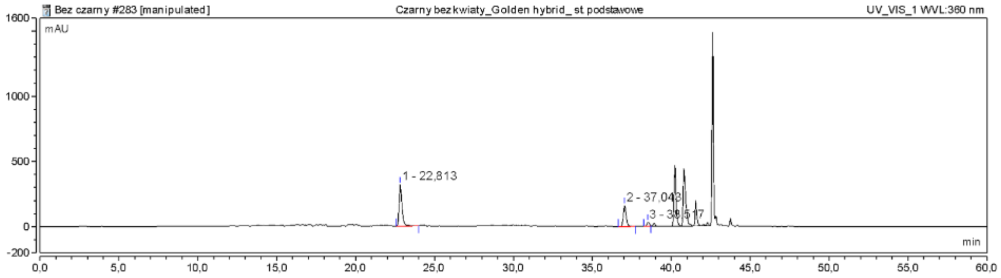
Bez czarny #283 [manipulated]
Czarny bez kwiaty_Golden hybrid_st podstawowe
UV_VIS_1 WVL:360 nm
mAU
1 - 22,813
2 - 37,043
3 - 38,517
min

e.

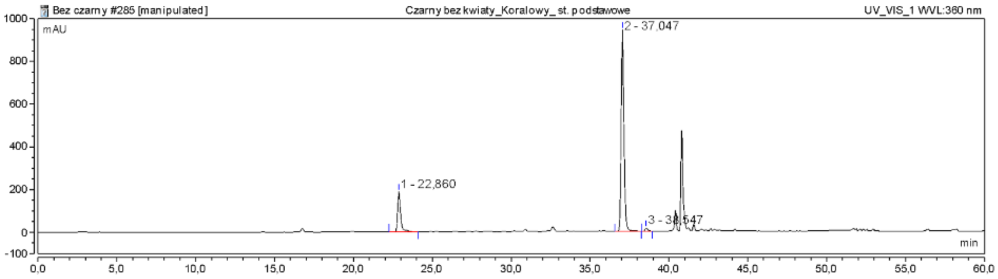
Bez czarny #285 [manipulated]
Czarny bez kwiaty_Koralowy_ st. podstawowe
UV_VIS_1 WVL:360 nm
mAU
1 - 22,860
2 - 37,047
3 - 38,547
min

f.

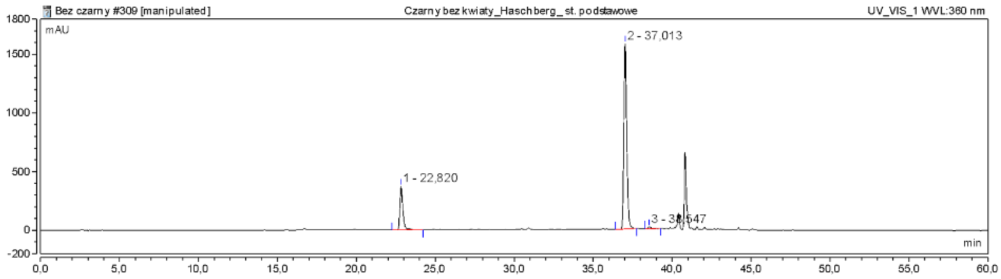
Bez czarny #309 [manipulated]
Czarny bez kwiaty_Haschberg_ st. podstawowe
UV_VIS_1 WVL:360 nm
mAU
1 - 22,820
2 - 37,013
3 - 38,547
min

g.

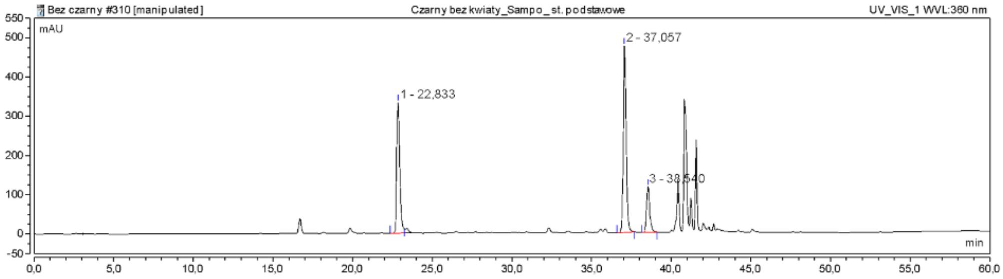
Bez czarny #310 [manipulated]
Czarny bez kwiaty_Sampo_ st. podstawowe
UV_VIS_1 WVL:360 nm
mAU
1 - 22,833
2 - 37,057
3 - 38,540
min

h.

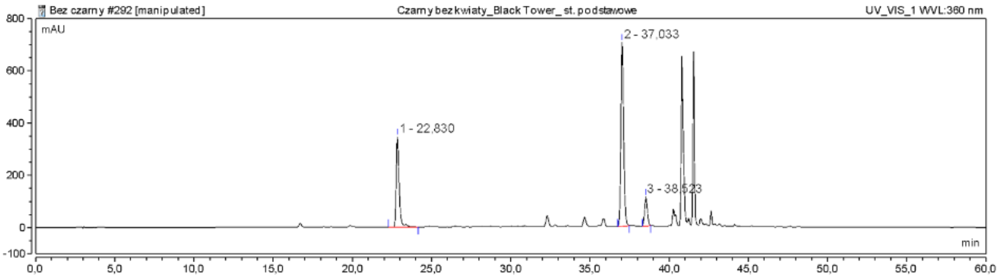
Bez czarny #292 [manipulated]
Czarny bez kwiaty_Black Tower_ st. podstawowe
UV_VIS_1 WVL:360 nm
mAU
1 - 22,830
2 - 37,033
3 - 38,523
min

i.

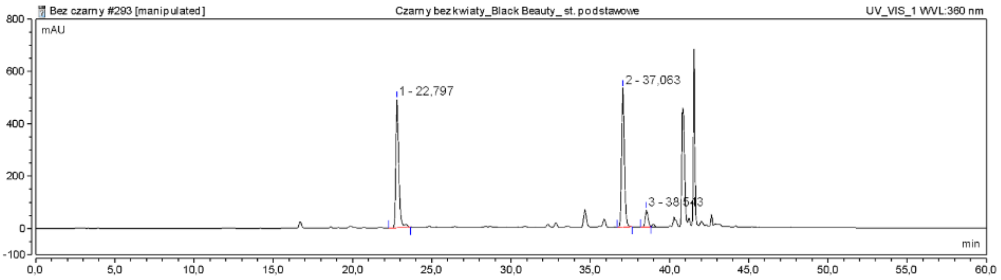
Bez czarny #293 [manipulated]
Czarny bez kwiaty_Black Beauty_ st. podstawowe
UV_VIS_1 WVL:360 nm
mAU
1 - 22,797
2 - 37,063
3 - 38,543
min

j.

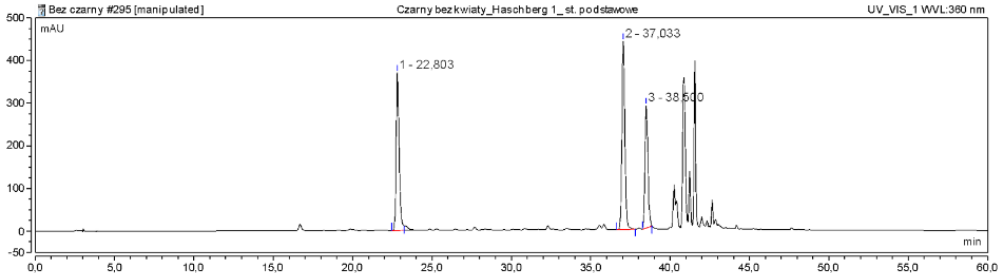
Bez czarny #295 [manipulated]
Czarny bez kwiaty_Haschberg 1_ st. podstawowe
UV_VIS_1 WVL:360 nm
mAU
1 - 22,803
2 - 37,033
3 - 38,500
min

k.

l.

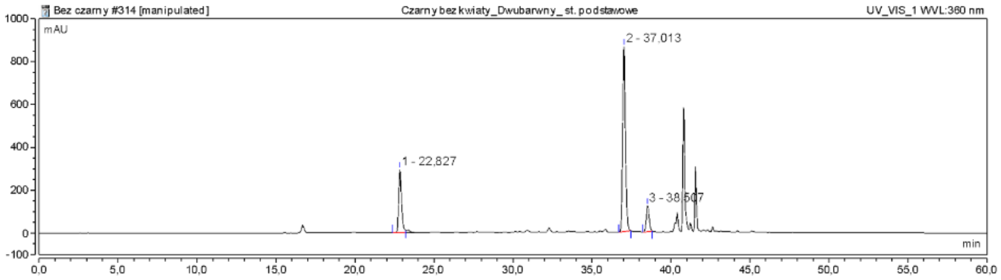

m.

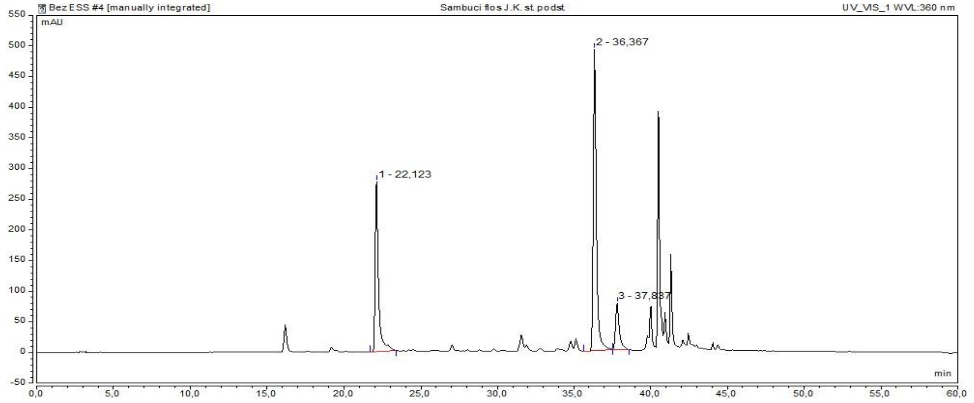

Figure S1. Chromatograms of 45% hydroalcoholic extracts from the flowers of each tested elderberry cultivars: Samyl (a), Samyl 1 (b), Obelisk (c), Sambo (d), Golden Hybrid (e), Red Elderberry (f), Haschberg (g), Sampo (h), Black Tower (i), Black Beauty (j), Haschberg 1 (k), Bez dwubarwny (l) and Wild elderberry (m). Peaks correspond to chlorogenic acid (1), rutin (2), and isoquercitrin (3).

Table S1. Correlation matrix

Variable	CGA	Rutin	Isoquer.	TPC	TFC	DPPH	CUPRAC	Gluc.I.	Hyal
CGA	1.0000	0.0049	0.2512	0.8137	0.1267	0.9343	0.8792	0.8034	0.4904
Rutin	0.0049	1.0000	-0.4511	-0.3006	0.6235	0.1987	-0.1639	0.0674	-0.7358
Isoquer.	0.2512	-0.4511	1.0000	0.3775	-0.2929	0.2009	0.3002	0.1196	0.5663
TPC	0.8137	-0.3006	0.3775	1.0000	0.0060	0.7555	0.8620	0.7209	0.7175
TFC	0.1267	0.6235	-0.2929	0.0060	1.0000	0.4139	0.2370	0.4512	-0.5009
DPPH	0.9343	0.1987	0.2009	0.7555	0.4139	1.0000	0.8434	0.8377	0.2887
CUPRAC	0.8792	-0.1639	0.3002	0.8620	0.2370	0.8434	1.0000	0.9352	0.6371
Gluc.I	0.8034	0.0674	0.1196	0.7209	0.4512	0.8377	0.9352	1.0000	0.4560
Hyal	0.4904	-0.7358	0.5663	0.7175	-0.5009	0.2887	0.6371	0.4560	1.0000
